# Morphotype-specific calcium signaling in human microglia

**DOI:** 10.1186/s12974-024-03169-6

**Published:** 2024-07-17

**Authors:** Sofia Nevelchuk, Bianca Brawek, Niklas Schwarz, Ariel Valiente-Gabioud, Thomas V. Wuttke, Yury Kovalchuk, Henner Koch, Anke Höllig, Frederik Steiner, Katherine Figarella, Oliver Griesbeck, Olga Garaschuk

**Affiliations:** 1 Department of Neurophysiology, Institute of Physiology, Eberhard Karls University of Tübingen, Keplerstr. 15, 72074 Tübingen, Germany; 2 Department of Neurology and Epileptology, Hertie Institute for Clinical Brain Research, University of Tübingen, Tübingen, Germany; 3 Tools for Bio-Imaging, Max-Planck-Institute for Biological Intelligence, Martinsried, Germany; 4 Department of Neurosurgery, University of Tübingen, Tübingen, Germany; 5 Department of Epileptology, Neurology, RWTH Aachen University Hospital, Aachen, Germany; 6 Department of Neurosurgery, RWTH Aachen University, Aachen, Germany; 7 Present Address: Department of Anesthesiology, Critical Care and Pain Medicine, University of Texas Health Science Center at Houston, Houston, TX USA

**Keywords:** Human microglia, In vitro human brain tissue model, Native microglial microenvironment, Morphotypes of resident microglia, microRNA-9-assisted labeling

## Abstract

**Background:**

Key functions of Ca^2+^ signaling in rodent microglia include monitoring the brain state as well as the surrounding neuronal activity and sensing the danger or damage in their vicinity. Microglial Ca^2+^ dyshomeostasis is a disease hallmark in many mouse models of neurological disorders but the Ca^2+^ signal properties of human microglia remain unknown.

**Methods:**

We developed a novel genetically-encoded ratiometric Ca^2+^ indicator, targeting microglial cells in the freshly resected human tissue, organotypically cultured tissue slices and analyzed in situ ongoing Ca^2+^ signaling of decades-old microglia dwelling in their native microenvironment.

**Results:**

The data revealed marked compartmentalization of Ca^2+^ signals, with signal properties differing across the compartments and resident morphotypes. The basal Ca^2+^ levels were low in ramified and high in ameboid microglia. The fraction of cells with ongoing Ca^2+^ signaling, the fraction and the amplitude of process Ca^2+^ signals and the duration of somatic Ca^2+^ signals decreased when moving from ramified via hypertrophic to ameboid microglia. In contrast, the size of active compartments, the fraction and amplitude of somatic Ca^2+^ signals and the duration of process Ca^2+^ signals increased along this pathway.

**Supplementary Information:**

The online version contains supplementary material available at 10.1186/s12974-024-03169-6.

## Background

Microglia are the principal immune cells of the central nervous system (CNS), which are implicated in virtually all physiological (e.g., development, synaptic transmission, plasticity and sleep) and pathological (e.g., traumatic injury, glioma and neurodegenerative or autoimmune diseases) processes of the CNS [1, 2]. A key aspect of microglial function is the ability to monitor their microenvironment and detect dyshomeostasis by sensing DAMPs (damage-) or PAMPs (pathogen-associated molecular patterns). For this purpose, they express a plethora of genes encoding different membrane receptors, the sum of which is referred to as microglial sensome [3]. In many cases, activation of those receptors leads to an increase in the intracellular free Ca^2+^ concentration ([Ca^2+^]_i_). Such transient changes in [Ca^2+^]_i_ link the sensor and effector functions of microglia by triggering the generation/release of cytokines and other inflammatory factors (e.g., reactive oxygen species), proliferation, differentiation, migration and phagocytosis [4, 5]. In mice, microglial Ca^2+^ transients are spatially compartmentalized and located in subcellular domains involved in a given (patho)physiological function. The normal function of cortical neural networks, for example, is accompanied by infrequent Ca^2+^ transients in microglial processes, with the frequency of process transients increasing dramatically during neural network hyper- or hypoactivity [6]. Consistently, somatic Ca^2+^ transients are rare under homeostatic conditions but much more frequent during (neuro)inflammation, injury or damage [7–11]. Although under homeostatic conditions the mouse microglial sensome broadly resembles that of humans [12–14], knowledge about the Ca^2+^ dynamics of human microglia is scarce and so far restricted to cultured primary microglia (e.g., [15]) or cultured human induced pluripotent stem cell (iPSC)-derived microglia-like cells (e.g., [16–20]).

Moreover, the morphological appearance of microglia in the intact human brain is indicative of a higher state of alertness compared to the mouse microglia [21, 22]. Whereas in mice the vast majority of cortical microglia, for example, show a ramified phenotype, microglia in the human cortex also appear in a primed, reactive, or amoeboid morphology. The reason for the dominance of ramified microglia over other morphotypes in mice remains unclear. This might be due to the specific pathogen-free environment of laboratory animals, which differs a lot from the typical environment of a human individual, or to the fact that the turnover rate of microglia in the human CNS is lower than that in the mouse CNS [23, 24]. This discrepancy, however, poses the question of whether human and mouse microglia differ in terms of their Ca^2+^ signaling.

To answer this question, we studied the Ca^2+^ signaling of human microglia residing in the spare cortical tissue obtained during glioblastoma/astrocytoma surgery or hippocampectomy (Table 1). The cells within the sliced tissue were transduced with lentiviral vectors encoding a newly developed ratiometric Ca^2+^ indicator mCyRFP1-CaNeon and the slices were organotypically cultured in human cerebrospinal fluid (hCSF). This preparation preserves well the cytoarchitecture and electrophysiological properties of excitatory and inhibitory neurons for several weeks [25, 26].

**Table 1:** Tissue used for organotypic slice culturing

Patient no.	Age at surgery (y)	Sex	Resected brain area	Surgery cause	DIV*	Number of cells analyzed
1	56	F	Left temporal cortex	Glioblastoma, WHO grade IV	9	110 (Fig. 1C, D)
2	54	M	Left temporal cortex	Anaplastic astrocytoma, WHO grade III	9	5 (Fig. 2D) 2 (Fig. 2D, 3, 4, 5, S5)
3	45	F	Right temporal cortex	Diffuse astrocytoma, WHO grade II	7–13	17 (Figs. 3, 4, 5, S5)
4	37	M	Right frontal cortex	Astrocytoma, WHO grade III	7–12	4 (Fig. 2D) 11 (Figs. 2D, 3, 4, 5, S5) 14 (Figs. 3, 4, 5, S5)
5	11	F	Right temporal cortex	Hippocampal sclerosis right	7–9	21 (Figs. 3, 4, 5, S5)
6	41	M	Right parietal cortex	Intraventricular glioneuronal tumor (high grade)	7–9	16 (Figs. 3, 4, 5, S5)

*DIV days in vitro

**Fig. 1:**
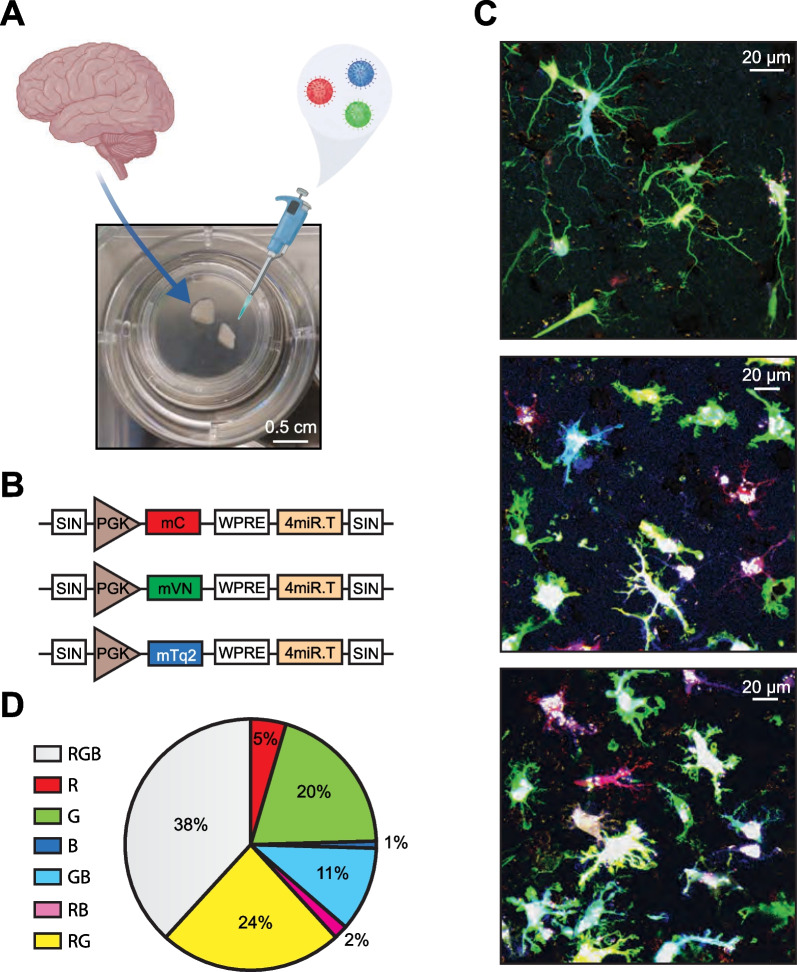
miR-9-assisted labeling of human microglia. **A** Schematics illustrating preparation, culturing and RGB labeling of human organotypic brain slices. **B** Scheme of the miR-9-regulated viral constructs inducing expression of mCherry (red), mVenus (green) and mTurquoise (blue) in microglia. **C** Maximum intensity projection (MIP) images (2–20 µm depth, here and below step size 1 µm) showing RGB-labeled human microglia in organotypic slices. **D** Pie chart showing the fractions of different colors in the RGB-labeled microglial population (n = 110 cells)

**Fig. 2:**
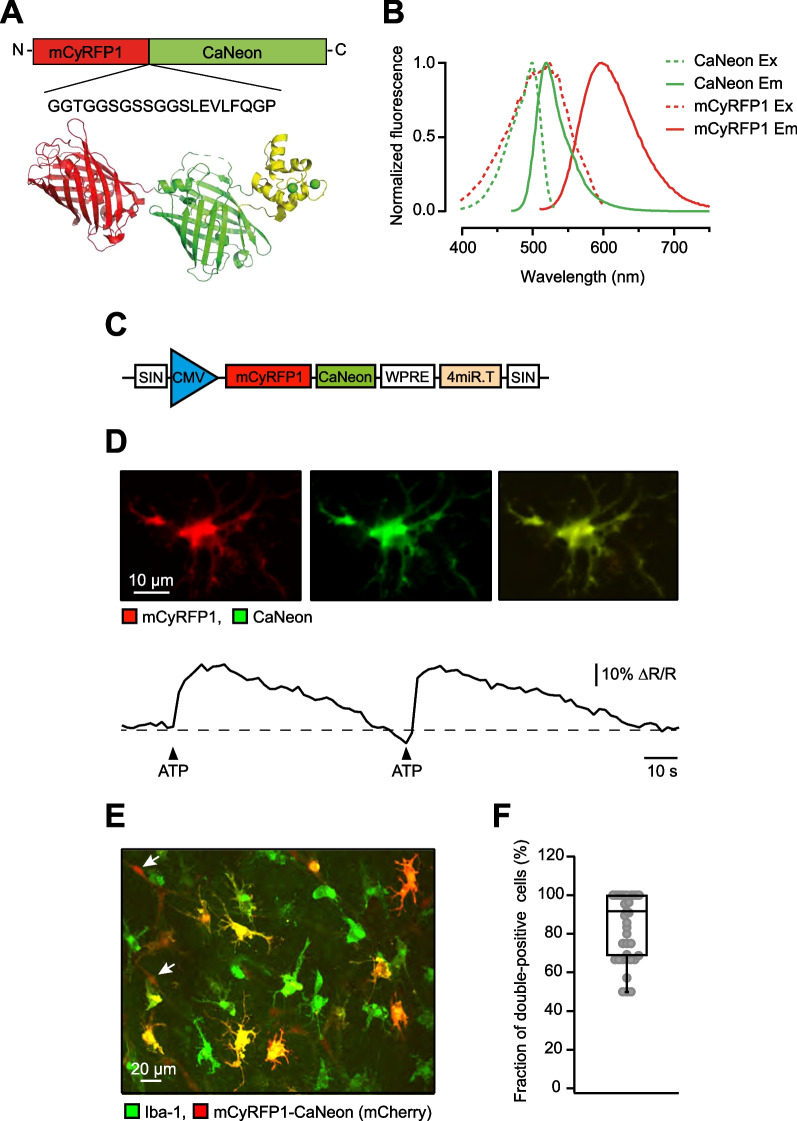
Functional properties of the new ratiometric Ca^2+^ sensor mCyRFP1-CaNeon. **A** Schematics of the sensor design (see Materials and methods for details). **B** The excitation and emission spectra of purified mCyRFP1 (red) and CaNeon (green). Note that the excitation spectra show significant overlap, enabling the efficient one-photon excitation of the fluorophore by a single light source, and the emission spectra can be well separated. **C** Scheme of the miR-9-regulated viral construct for expressing mCyRFP1-CaNeon in in situ microglia. **D** Sample Ca^2+^ transients (lower panel) evoked by pressure application (12 psi, 200 ms) of 5 mM ATP (dissolved in the standard pipette solution: 150 mM NaCl, 2.5 mM KCl, 10 mM HEPES, pH 7.4) to a mCyRFP1-CaNeon-expressing cell (upper panel). **E** MIP (6–16 µm depth) image of a fixed organotypic slice, labeled with antibodies against a microglia/macrophage marker Iba-1 (green) and mCyRFP1-CaNeon (red). **F** Box plot showing the fraction of double-positive cells among the cells, positive for mCyRFP1-CaNeon (n = 254 mCyRFP1-CaNeon -positive cells, 41 FOVs)

**Fig. 3:**
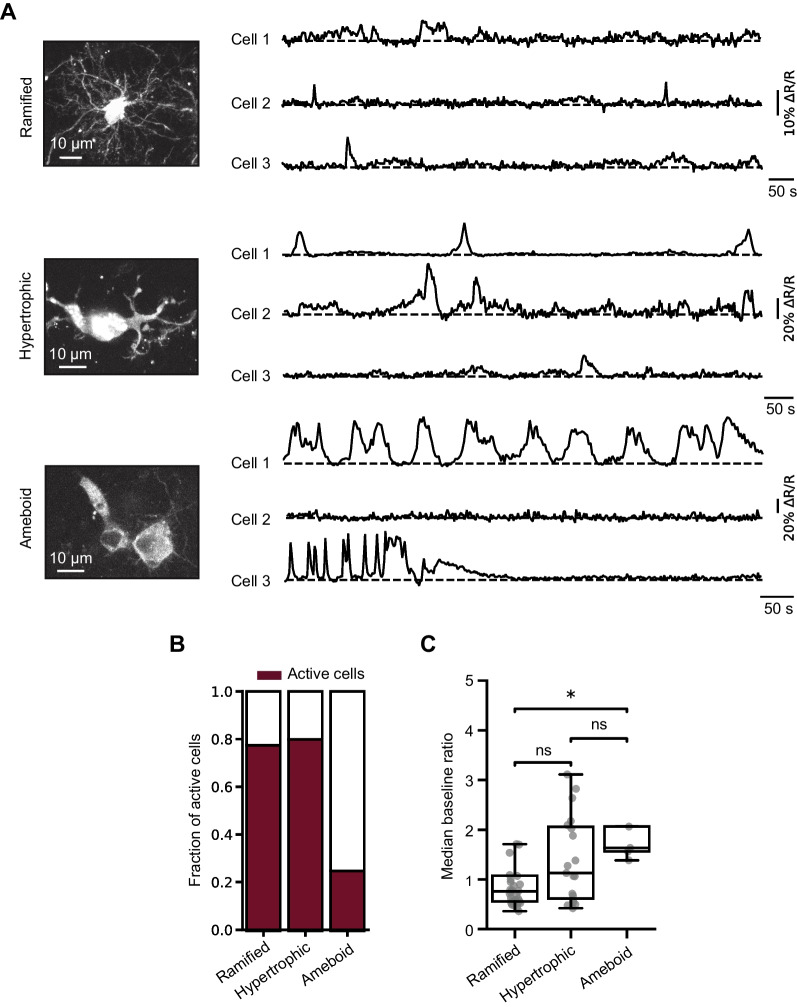
Ongoing Ca^2+^ signaling in microglia of different morphology. **A** MIP images showing examples of mCyRFP1-CaNeon-positive microglia with ramified, hypertrophic, and amoeboid morphology (left; 8–32 µm, 7–30 µm, and 3–16 µm depth, respectively) as well as whole-cell ΔR/R traces, recorded from these (Cell 1 of each morphotype) as well as other cells during an ~ 15-min-long recording period (right). **B** Bar graph summarizing the fractions of active cells of different morphologies (n = 38 ramified, 25 hypertrophic and 18 ameboid microglia). **C** Box plot illustrating the distributions of the median (per cell) basal ratios of ramified, hypertrophic and ameboid microglia (P = 1.2*10^–2^ for comparison between ramified and ameboid microglia, Kruskal–Wallis test; n = 31, 20, 5 active cells, respectively)

**Fig. 4:**
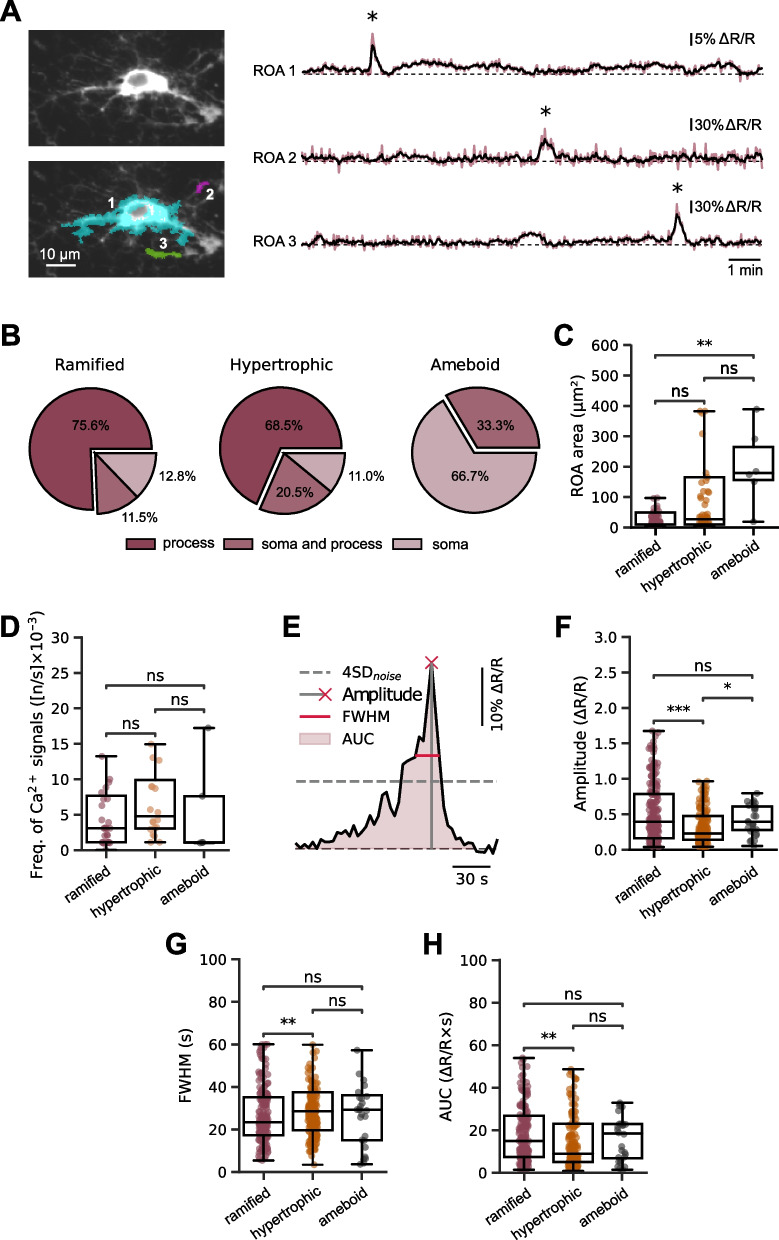
Subcellular compartmentalization of ROAs in human microglia. **A** Left: 4D average intensity projection (24 µm depth) of a mCyRFP1-CaNeon-expressing ramified microglial cell alone (upper panel) and with the overlayed sample ROAs, shown in different colors (lower panel). Right: spontaneous ongoing Ca^2+^ signals (asterisks) recorded from ROAs, labeled with the respective number in the lower left panel. **B** Pie charts showing the spatial distribution of ROAs in ramified (left panel), hypertrophic (middle panel), and amoeboid (right panel) microglia. **C** Box plots showing the morphotype-specific distributions of ROA areas. ROAs were significantly smaller in ramified compared to amoeboid microglia (P = 10^–3^; here and below: the Kruskal–Wallis test followed by the Holm-Bonferroni post hoc test for multiple comparisons). **D** Box plots illustrating the frequencies per cell of Ca^2+^ transients in ramified, hypertrophic and ameboid microglia (n = 31, 20, 5 cells, respectively). **E** Schematic, defining the parameters of Ca^2+^ transients analyzed in this study. **F–H** Box plots showing amplitudes (**F**; P = 3.4*10^–4^ and 0.02 for comparison of ramified to hypertrophic and hypertrophic to ameboid microglia, respectively), FWHM (**G**; P = 8.7*10^–3^ for comparison of ramified to hypertrophic microglia) and AUC (**H**; P = 3.3*10^–3^ for comparison of ramified to hypertrophic microglia) of Ca^2+^ transients for microglia of different morphologies

**Fig. 5:**
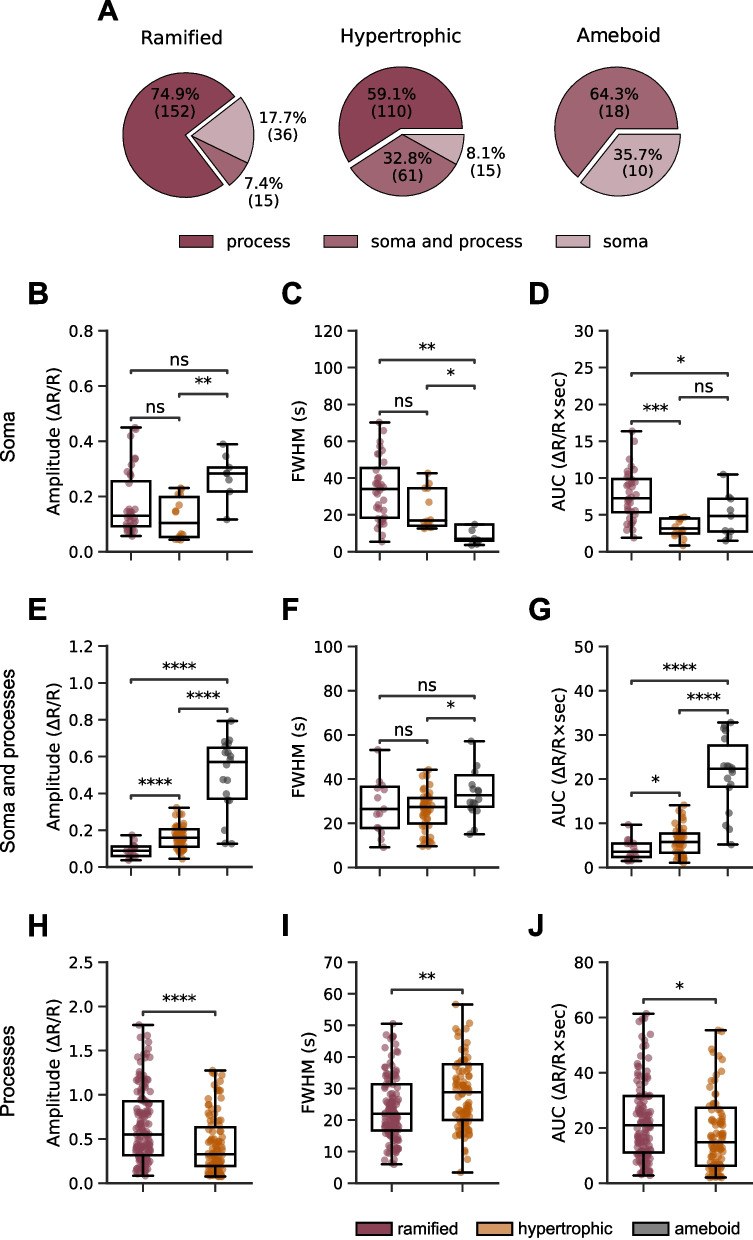
Characteristics of microglial Ca^2+^ transients in different subcompartments. **A** Pie charts showing the distribution of Ca^2+^ transients among different subcompartments in ramified (left panel), hypertrophic (middle panel), and amoeboid (right panel) human microglia. **B**–**D** Box plots comparing amplitude (**B**; P = 6*10^–3^ for comparison of hypertrophic to ameboid microglia (here and below: the Kruskal–Wallis test followed by the Holm-Bonferroni post hoc test for multiple comparisons)), FWHM (**C**; 1.4*10^–3^ and 0.01 for comparison of ramified to ameboid and hypertrophic to ameboid microglia, respectively), and AUC (**D**; P = 0.03 and 2.2*10^–4^ for comparison of ramified to ameboid and ramified to hypertrophic microglia, respectively) of somatic Ca^2+^ transients in ramified, hypertrophic and amoeboid microglia. **E–G** Box plots comparing amplitude (**E**; P = 4.8*10^–5^, 2.1*10^–6^ and 4.6*10^–7^ for comparison of ramified to hypertrophic, ramified to ameboid and hypertrophic to ameboid microglia, respectively), FWHM (**F**; P = 0.01 for comparison of hypertrophic to ameboid microglia), and AUC (**G**; P = 3.7*10^–2^, 4.4*10^–6^ and 1.8*10^–8^ for comparison of ramified to hypertrophic, ramified to ameboid and hypertrophic to ameboid microglia, respectively) of Ca^2+^ transients in soma and processes of ramified, hypertrophic and amoeboid microglia. **H–J** Box plots comparing amplitude (**H**; P = 3.1*10^–5^), FWHM (**I**; P = 1.3*10^–3^), and AUC (**J**; P = 0.02) of process Ca^2+^ transients of ramified and hypertrophic microglia

## Materials and methods

### Tissue specimen

Organotypic cortical slices were prepared from the cortical tissue, surgically resected to gain access to the pathology (Table 1). Approval (# 772/2021BO2) of the ethics committee of the University of Tübingen as well as written informed consent was obtained from all patients, whose resected tissue was used in this study.

Immunohistochemical data shown in Supplementary Fig. 4 were obtained from the cortical tissue, surgically resected to gain access to the pathology, and fixed immediately after resection (Table 2). Approval (EK067/20) of the ethics committee of the RWTH Aachen University as well as written informed consent for tissue donation and approval of the use of human tissue for scientific purposes and all related experimental procedures was collected before the commencement of the study.

**Table 2:** Tissue used for immediate fixation

Patient no.	Age at surgery (y)	Sex	Resected brain area	Surgery cause
1	74	F	Right frontal cortex	Metastasis (non-small cell lung cancer)
2	34	M	Left orbitofrontal cortex	Diffuse glioneuronal tumor with oligodendroglioma-like features and nuclear clusters
3	75	F	Right frontal cortex	Glioblastoma
4	73	M	Left temporal cortex	Glioblastoma

### Slice preparation and culturing

Slice preparation and culturing were performed as detailed previously [25, 26]. Briefly, the cortex was resected, microdissected, and immediately transferred into the ice-cold artificial cerebrospinal fluid (aCSF) of the following composition (in mM): 110 choline chloride, 26 NaHCO_3_, 10 D-glucose, 11.6 Na-ascorbate, 7 MgCl_2_, 3.1 Na-pyruvate, 2.5 KCl, 1.25 NaH_2_PO_4_, und 0.5 CaCl_2_, pH 7.4, when bubbled with 95% O_2_, 5% CO_2_. The tissue was kept submerged in cool and oxygenated aCSF at all times. Slices (thickness 250–350 µm) were cut using a vibratome (Microm HM 650 V, Thermo Fisher Scientific Inc, USA) and transferred onto culture membranes for cultivation. For the first hour following the slicing procedure, the slices were cultured in 1.5 ml neural stem cell media (48% DMEM/F-12 (Life Technologies), 48% Neurobasal (Life Technologies), 1 × N-2 (Capricorn Scientific), 1 × B-27 (Capricorn Scientific), 1 × Glutamax (Life Technologies), 1 × NEAA (Life Technologies), 20 mM HEPES) before changing to 1.5 ml hCSF per well without any supplements. For transduction with lentiviral vectors, encoding either a combination of 3 different fluorophores (mCherry, mVenus, mTurquoise) or the novel genetically encoded Ca^2+^ indicator mCyRFP1-CaNeon (see below), 1–2 µl of the respective virus or virus mixture solution was added to the surface of each section within the first 24 h after resection. Thereafter, the slices were cultured for 9 ± 1.5 days on culture membranes (uncoated 30 mm Millicell-CM tissue culture inserts with 0.4 mm pores, Merck Millipore, Germany) in six-well dishes (BD Biosciences) each containing 1.5 ml of hCSF. The dishes were kept in an incubator (Thermo Scientific) running at 37 °C, 5% CO_2_ and 100% humidity and hCSF was replaced every 3 days. When taken out of a six-well dish, each slice was imaged for 3–4 h and then fixed with 4% formaldehyde in PBS as described below.

Human CSF (for ionic composition see ref. [27]) was collected from lumbar punctures for pressure relief of patients with idiopathic intracranial hypertension or diagnostics of normal pressure hydrocephalus. Written and informed consent was obtained for every patient under the approval of the local ethics committee (# 772/2021BO2). Thereafter the hCSF was centrifuged at 4 ºC and 2000 g for 10 min, filtered through the sterile filter (Ø 0.2 μm), the osmolarity (280 ± 20 mOsm/l) was controlled and the hCSF was frozen at -80 ºC in 10 ml aliquots until further use (for details see [25, 28]).

### Novel Ca^2+^ indicator CaNeon

When developing the ultralow affinity Ca^2+^ sensor GreenT-EC [29], we also obtained the crystal structure of an intermediate variant named NRS 1.2 (PDB 8COT). Starting from NRS 1.2 we developed CaNeon via several iterative rounds of optimizations and screenings. Compared to NRS1.2, CaNeon incorporates the following additional mutations: C159Y, D198G, G248D, S255C, I258R. These amino acid changes improved the Ca^2+^ binding properties of the sensor, as well as its expression in mammalian cells. The full protein sequence of CaNeon is displayed in Supplementary Fig. 1A. For designing a ratiometric version of the sensor, we used the red fluorescent protein mCyRFP1 as a reference fluorophore. The spectroscopic properties of mCyRFP1 allow simultaneous one- and two-photon-based excitation of both mCyRFP1 and CaNeon with an efficient spectral separation of the emission channels [30]. The mCyRFP1 was cloned at the N-terminus of CaNeon using a 20 amino acid-long flexible hydrophobic linker (GGTGGSGSSGGSLEVLFQGP, Fig. 2A). All cloning steps were done using the homology-based SliCE method [31]. In the absence of Ca^2+^, the fluorescence of CaNeon is low (Supplementary Fig. 1B). The binding of Ca^2+^ ions causes conformational changes in CaNeon, resulting in a strong increase in fluorescence. The spectroscopic in vitro properties of CaNeon and the fusion protein mCyRFP1-CaNeon are summarized in Supplementary Fig. 1D.

### Protein purification and biophysical characterization of CaNeon

His-tagged proteins were expressed in E.coli BL21 (Invitrogen) overnight at 37 °C in 50 mL auto-inductive Luria–Bertani medium, supplemented with 0.05% D-( +)- glucose (w/v), 0.2% lactose (w/v), 0.6% glycerol (v/v). Bacteria were harvested by centrifugation (4 °C, 10 min, 6000 g) and re-suspended in 10 mL resuspension buffer (20 mM Na_2_PO_4_, 300 mM NaCl, 20 mM imidazole; Sigma Aldrich) supplemented with protease inhibitors (4 μM PMSF, 20 μg/mL Pepstatin A, 4 μg/mL Leupeptin; Sigma Aldrich), 5 μg/mL DNase and 10 μg/mL RNase (Sigma Aldrich). Resuspended bacteria were lysed through sonication on ice for 7 min (80% of the time on; Bandelin Sonoplus). Insoluble components were removed through centrifugation (4 °C, 30 min at 20,000 g). The supernatant was incubated with 150 μL 6% (v/v) Nickel-IDA agarose bead suspension (Jena Bioscience) for 1 h at 4 °C under mild agitation. Agarose beads were collected in 1 mL propylene gravity flow columns (Qiagen) and washed with 10 mL resuspension buffer. The proteins were collected using 800 μL elution buffer (20 mM Na_2_PO_4_, 300 mM NaCl, 300 mM imidazole; Sigma Aldrich) and dialyzed against MOPS buffer (30 mM MOPS (3-morpholinopropane-1-sulfonic acid), 100 mM KCl, pH 7.2) for further measurements.

The ratio change (ΔR/R_0_) of purified mCyRFP1-CaNeon was determined by measuring the fluorescence at 520 and 650 nm upon excitation at 488 nm in MOPS buffer supplemented with 10 mM EGTA or 0.2 mM Ca^2+^ (Supplementary Fig. 1D). The molar extinction coefficients (EC) were determined via quantifying protein concentrations using the absorption of the denatured chromophore at 452 nm (with an EC of 44 mM^−1^ cm^−1^). Proteins were prepared in MOPS buffer supplemented with 0.2 mM CaCl_2_ and the absorbance spectrum was acquired before and after the addition of NaOH to a final concentration of 0.1 M. The quantum yield of CaNeon in mCyRFP1-CaNeon was determined relative to mNeonGreen using the slope method, measuring the absorbance and emission spectra of serial dilution of the proteins. For the quantum yield and extinction coefficient measurements, the formation of CyRFP1 chromophore was prevented by incorporating the mutation Y68C. This allowed us to determine the structural effect of the N-terminal tag on CaNeon without the spectral interference of mCyRFP1. The brightness was calculated as extinction coefficient ˣ quantum yield.

The Ca^2+^ affinity of the sensors was determined using MOPS buffer supplemented with 10 mM EGTA and 1 mM Mg^2+^ by increasing concentrations of Ca^2+^ as previously described [31, 32]. The dissociation constant (Kd) values were determined by plotting the log10 values of the free Ca^2+^ concentrations against the corresponding ΔF/F_0_ or ΔR/R_0_ values (normalized to the response at 39.8 μM Ca^2+^) and fitting a sigmoidal curve to the plot. The kinetic rates of the Ca^2+^ indicators were measured in a Varian Cary Eclipse fluorescence spectrophotometer fitted with an Applied Photophysics RX pneumatic drive unit. For obtaining the macroscopic off-rate constant (K_off_), two stock solutions were prepared as follows: a Ca^2+^-saturated indicator solution (30 mM MOPS, 1 mM CaCl_2_, 2 mM MgCl_2_, 100 mM KCl, ∼ 0.2–1 μM indicator, pH 7.2) and a BAPTA solution (30 mM MOPS, 100 mM KCl, 20 mM BAPTA, pH 7.2). The stopped-flow experiment was carried out at room temperature (∼23 °C) and the two solutions were mixed with an injection pressure of 3.5 bar. Excitation was set to 480 nm and emission was detected at 520 nm. The decay time (τ, s) was determined by fitting a double-exponential curve to the fluorescence response using GraphPad Prism version 9.5.1 for Windows (GraphPad Software). Macroscopic on-rate kinetics (K_obs_) were obtained both for CaNeon and mCyRFP1-CaNeon by mixing the Ca^2+^-free buffer containing the protein (30 mM MOPS, 100 mM KCl, 1 mM MgCl_2_, 10 mM EGTA, ∼ 0.2–1 μM indicator, pH 7.2) and solutions containing increasing concentrations of CaCl_2_ (30 mM MOPS, 100 mM KCl, 8–20 mM CaCl_2_, 1 mM MgCl_2_, 10 mM EGTA, ∼ 0.2–1 μM indicator, pH 7.2). Concentrations of free Ca^2+^ were calculated using WEBMAXC STANDARD.

For measuring the pKa of the sensors a series of MOPS/MES (2-N-morpholino-ethane sulfonic acid) buffered solutions, supplemented with 1 mM Ca^2+^, were prepared. The pH values were adjusted in 0.5 pH steps from pH 5.5 to pH 8.5 using NaOH and HCl. In a bottom 96 well plate, triplicates of 200 μl of buffer containing 0.5–1 μM of protein were prepared for each pH value and all emission spectra were recorded. The relative fluorescence values at the emission maximum were plotted against the pH values and a sigmoidal fit was applied.

### Creation of the viral vector carrying mCyRFP1-CaNeon

We used the LV.Twitch-2B.miR-9.T construct containing the cytomegalovirus (CMV) promoter and four microRNA-9 target sequences [10] as the parental vector to produce the LV. mCyRFP1-CaNeon.miR-9.T. We replaced Twitch-2B with mCyRFP1-CaNeon using a homologous recombination-based assembly molecular cloning approach. For this purpose, we propagated mCyRFP1-CaNeon by PCR amplification from a donor vector using primers that contain both mCyRFP1-CaNeon specific sequences and the flanking regions (15 nucleotides in length) located exactly up- and down-stream of the Twitch-2B sequence in the parenteral vector (forward primer: 5ʹ-CTCTACTAGAGGATCCGCCACCATGGTGAGCAAGGGC-3ʹ, reverse primer: 5ʹ-GAGGTTGATTGTCGACTCAGTGGTATTTGTGAGCCAGGG-3ʹ). The PCR product was digested with Dpn I, affinity-purified, and reserved for assembly molecular cloning. Then, the parental vector LV.Twitch-2B.miR-9.T was digested with the restriction enzymes BamH I and Sal I to remove the Twitch-2B sequence. The homologous recombination reaction was performed at a 1:2 ratio with 200 ng of the digested parenteral vector using the NEBuilder® HiFi DNA Assembly Cloning Kit from New England BioLabs.

### Production of microRNA-9-regulated lentiviral vectors

MicroRNA-9 (miR-9) is a microRNA that promotes the degradation of mRNA with a specific complementary sequence. Because in mice miR-9 is expressed in virtually all CNS cells except microglia, it can be used for cell type-specific labeling of microglia [10, 33, 34]. This cell type specificity, however, relies on the interaction between the endogenous miR-9 and the exogenous substrate and thus can be overridden, if the concentration of exogenous mRNA is high or the concentration of endogenous miR-9 is low.

Production of miR9-regulated lentiviral vectors was described previously [34, 35]. In brief, cell-free supernatants containing viral particles were produced by transient transfection of HEK293T packaging cells with the respective lentiviral construct along with the packing plasmids psPAX2 and pMD2G, following standard procedures. After 48 h the virus-containing culture supernatant was collected, filtered through a 0.45 µm pore-sized filter to remove cell debris and concentrated by centrifugation at 100,000 g for 2 h at 4 °C (Thermofisher WX Ultra80 centrifuge, Waltham, MA, USA). Pellets were resuspended in sterile PBS and stored at − 80 °C.

Four different miR-9-regulated lentiviral constructs were used in this study. Constructs containing the PGK promotor (Fig. 1B) enabled the expression of 3 different fluorophores (mCherry, Venus, or mTurquoise2) and the stochastic combination thereof [34]. The LV.mCyRFP1-CaNeon.miR-9.T construct contained a CMV promotor (Fig. 2C) and was used to label human microglia with a ratiometric Ca^2+^ indicator mCyRFP1-CaNeon. For this study, viral suspensions with 2.5–4 × 10^8^ virus particles per ml were used.

### Two-photon imaging

Two-photon imaging was performed using a laser scanning microscope (Olympus Fluoview 1000 or Olympus Fluoview 300, Olympus, Tokyo, Japan) equipped with a 40 × water-immersion objective (0.80 NA, Nikon, Tokyo, Japan) and coupled to a tunable titanium-sapphire laser (690–1040 nm excitation wavelength; MaiTai DeepSee, Spectra Physics, Santa Clara, CA, USA). During imaging the human slices were continuously perfused with Ringer's solution (125 mM NaCl, 4.5 mM KCl, 26 mM NaHCO_3_, 1.25 mM NaH_2_PO_4_, 2.5 mM CaCl_2_, 1 mM MgCl_2_, and 20 mM glucose) at 32 °C and pH 7.4, when bubbled with 95% O_2_ and 5% CO_2_.

mTurquoise and mCherry were excited at 800 nm and mVenus at 990 nm. The emitted light was split by a 570 nm dichroic mirror and filtered with SP 570 nm or BP 630/92 nm, respectively. mCyRFP1-CaNeon was excited at 930 nm and a 570 nm dichroic mirror was utilized to split the emitted light into two separate channels for CaNeon (SP 570 nm) and mCyRFP1 (BP 630/92 nm), respectively. Microglial Ca^2+^ transients were recorded from cells located 3–60 µm below the surface of the slice in 4D for 15 min with a resolution of 0.31 μm/pixel in the XY plane, Z step size of 2 µm and a sampling rate of 2 μs/pixel (0.3 to 0.5 Hz). 3D stacks were acquired at a spatial resolution of 0.31 μm/pixel and a Z step size of 1–2 μm.

Microglial morphotypes were defined based on visual inspection of high-resolution cell images using well-established morphological criteria [21, 36–38]. Cells with small cell bodies with long, thin, and highly branched processes were considered ramified, cells with enlarged cell bodies and thick, sparsely branched processes were considered hypertrophic, and cells with rounded cell bodies, few very short or no processes were considered ameboid (see Fig. 3A, Supplementary Fig. 4A, B).

### Ca^2+^ signal detection and analyses

Ca^2+^ signals were detected using the active voxels detection algorithm of the MATLAB (MathWorks) Begonia framework [39]. Within this framework, the fluorescence value $F$ of each pixel is converted into a binary time series, where pixels with values other than 0 represent events (Supplementary Fig. 2A). These events correspond to pixel grayscale values that exceed a user-determined threshold ${\tau }_{i}$, which is a function of the baseline grayscale values and the standard deviation of noise:

$${\tau }_{i}= {\widehat{\mu }}_{i}+\kappa \cdot {\widehat{\sigma }}_{i}$$

where $\kappa$ is an empirically obtained coefficient that determines the height of the threshold, ${\mu }_{i}$ is the baseline value of fluorescence for each pixel, and ${\sigma }_{i}$ is the standard deviation of noise [39]. Depending on the image quality and the fluorescence intensity of CaNeon, $\kappa$ values between 4 and 5 were chosen. The binary matrix of active pixels (Supplementary Fig. 2B) was then exported from MATLAB and further analyses were performed using a Python routine [40]. A map of all active pixels was obtained by summing up all time points that contain active pixels along the t-axis. As the value of active pixels is 1 and the value of inactive pixels is 0, the resulting image consists of the non-zero pixels that were active at any given point during the recording. Next, the active pixels were grouped into separate regions by connecting an active pixel to its immediate horizontal and vertical active neighbors. This procedure resulted in separate localized regions of activity (ROAs) that did not overlap in space throughout the entire registration (e.g., Fig. 4A).

To understand whether the given ROA was localized to the (i) soma, (ii) process or (iii) covered both soma and processes, the somatic area was identified by applying the Otsu threshold [41], which works by analyzing the histogram of pixel intensities in the averaged cell image and automatically selecting a threshold value that minimizes the variance within the two resulting groups: foreground (cell soma) and background (Supplementary Fig. 2C). Next, the somatic area determined in this way was overlayed with the ROA of interest to determine whether (i) the ROA is located within the somatic area (somatic ROA), (ii) outside the somatic area (process ROA) or (iii) covers the somatic area and largely extends beyond it (soma and process ROA; e.g., cyan ROA in Supplementary Fig. 2D).

Then, for both CaNeon and mCyRFP1 fluorescence, a mean intensity value for every frame in the area corresponding to each previously obtained ROA was calculated. The background-subtracted fluorescence ratio and the relative ratio change (ΔR/R) were calculated as follows:

$${\text{R}} = \left( {{\text{F}}_{{{\text{CaNeon}}}} - {\text{B}}_{{{\text{CaNeon}}}} } \right)/\left( {{\text{F}}_{{{\text{mCyRFP1}}}} - {\text{ B}}_{{{\text{mCyRFP1}}}} } \right)$$

$$\Delta {\text{R}}/{\text{R}}_{0} = \left( {{\text{R}} - {\text{R}}_{0} } \right)/{\text{R}}_{0}$$

where the background fluorescence (B) for both channels was calculated as the mean intensity of a 10 × 10 pixels region located outside the analyzed cell and R_0_ is the baseline value of R. The baseline was approximated with a third-order weighted polynomial function. The peaks of ΔR/R traces were detected by utilizing the Python scipy-signal module [42]. Only those peaks that exceeded the 4 × σ_noise_ threshold were considered.

### Immunohistochemistry

After imaging experiments, human organotypic slices were fixed with 4% formaldehyde in PBS for 2 h at 4 °C, washed in PBS, and permeabilized using 0.25% Triton X in PBS for 15 min. Antibody staining was performed with free-floating slices at room temperature. Slices were treated with a blocking solution (5% normal goat serum, 3% bovine serum albumin (BSA) and 1% Triton X-100 in PBS) for one hour and incubated overnight with the primary antibodies (rabbit-anti-Iba-1, 1:200; Wako, USA; rat-anti-mCherry, 1:2000, Thermo Fisher Scientific). After washing in PBS, the slices were incubated with Alexa Fluor (AF) 488- and AF-594-conjugated secondary antibodies (1:1000, Invitrogen, Waltham, MA, USA) for 2 h in darkness and later mounted on fluorescence-free Superfrost Plus microscope slides (Langenbrinck, Emmendingen, Germany) with Vectashield Mounting Medium (Vector Laboratories, Burlingame, CA, USA). Freshly resected cortical samples (Table 2) were fixed in 4% paraformaldehyde for 1 h at 4 °C and subsequently washed 3 × 15 min in PBS. The slices were transferred to a 15% sucrose solution for 90 min and then incubated in a 30% sucrose solution overnight. After washing the slices 3 × 15 min in PBS, they were incubated for 2 days at 4 °C with the rabbit-anti-Iba-1 antibody (1:1000, Wako, USA). After washing with PBS, the slices were incubated for 24 h at 4 °C with Alexa Fluor 488 secondary antibody (1:750, Thermo Fisher Scientific). Finally, the slices were washed with PBS and mounted using Fluoromount-G (Thermo Fisher Scientific).

For immunolabeling of cortical slices of NG2-DsRed transgenic mice (Tg(Cspg4-DsRed.T1)1Akik/J), we used the brain tissue from our biobank, which was previously isolated and fixed with 4% formaldehyde in PBS for 2 h at 4 °C, cryoprotected in 25% sucrose in PBS overnight at 4 °C, embedded in Tissue Tek (Sakura Finetek, Torrance, CA, USA) and stored at -80 °C. Antibody staining was performed with free-floating 50-μm-thick coronal sections at room temperature. The sections were rinsed in PBS, blocked in 1% bovine serum albumin (BSA) containing 0.3% Triton X-100 in PBS for 1 h at room temperature, followed by incubation with primary antibodies (rabbit-anti RFP, Rockland, 1:1500 and goat anti-PDGFRα, R&D Systems, 1:500) in 1% BSA, 0.1% Triton X-100 in PBS overnight. After washing in PBS, the slices were incubated with Alexa Fluor (AF) 488- and AF-594-conjugated secondary antibodies (1:1000, Invitrogen, Waltham, MA, USA) for 2 h in darkness; and later mounted on fluorescence-free Superfrost Plus microscope slides (Langenbrinck, Emmendingen, Germany) with Vectashield Mounting Medium (Vector Laboratories, Burlingame, CA, USA).

### Analyses of cell density, the distance to the nearest neighbor and the soma size

The "Spots" feature of the Imaris software (version 10.1.1; Bitplane, Oxford Instruments) was employed to detect and mark individual cells within 3D image stacks. Intensity thresholds were manually adjusted to ensure accurate detection of Iba-1-labeled microglia. Following cell identification, the statistics feature of Imaris was utilized to extract the distance to the nearest neighbors for each detected cell and the total number of cells. Cell density was then calculated by dividing the total number of cells by the stack volume.

For the reconstruction of microglial somata, cell bodies were manually identified and the surface creation feature with manual adjustment of thresholds was used to delineate the boundaries of microglial somata. Morphological and intensity segmentation features were employed to exclude adjacent extracellular components (e.g. lipofuscin grains). Cells were only included in the analyses if a clear separation from the surrounding extracellular material was possible. Following reconstruction, several parameters including 3 bounding box dimensions (i.e. cell diameters in X, Y, Z), the surface area and volume as well as sphericity were extracted from Imaris software.

### Statistical analyses and data presentation

Statistical tests were performed in Python using the Scipy library. The one-sample Shapiro–Wilk test was used to test for the normality of the data distribution and the Levene’s test was used to test for homoscedasticity. Comparisons of more than two independent variables were performed using the Kruskal–Wallis test followed by the Holm-Bonferroni post hoc test for multiple comparisons. All statistical tests were two-sided. The P values ≤ 0.05 were considered significant. If not otherwise indicated, data is presented as median ± interquartile range. Lines of boxes in box plots represent 25th and 75th, and whiskers 10th and 90th percentiles.

## Results

### New genetically encoded indicator for Ca^2+^ imaging in human microglia

To extend the methodology, previously developed for mice, to human tissue, we first transduced the organotypic cortical slices (Fig. 1A) with the 1:1:1 mixture of miR9-regulated lentiviral vectors encoding mCherry (red = R), mVenus (green = G), and mTurquoise2 (blue = B) by adding 1–2 µl of the virus mixture (Fig. 1B) to the surface of brain slices during culturing. Because of the high endogenous activity of miR9 in most brain cells except microglia, this approach favors the specific labeling of microglial cells [33, 34]. As shown in Fig. 1C, this protocol resulted in strong labeling of cells with microglia-like morphology. Due to the stochastic nature of viral transduction, individual cells expressed either one of the three fluorophores or any combination thereof (Fig. 1C and D).

Next, we set out to develop an indicator suited for assessing the [Ca^2+^]_i_ in human microglia. We opted for a ratiometric red/green indicator (Fig. 2A), which is less sensitive to movement artifacts and thus facilitates the identification of thin mobile processes, and engineered it to enable simultaneous fluorophore excitation in single- (Fig. 2B; excitation wavelength 488 nm) and two-photon modes.

Thus, we first constructed CaNeon, a non-ratiometric Ca^2+^ indicator consisting of the yellow-green fluorescent protein mNeonGreen [43] and a minimal Ca^2+^ binding domain derived from Troponin C (TnCmin) with only 2 Ca^2+^ binding sites (versus 4 in GCaMPs), thus lowering the buffer capacity of the indicator (Supplementary Fig. 1A–D). We started from GreenT-EC, which is tuned to monitor Ca^2+^ levels in interstitial fluids [29], and derived a variant that binds Ca^2+^ with high affinity while retaining large fractional fluorescence changes (Supplementary Fig. 1). We then conjugated this indicator to the red fluorescent protein mCyRFP1 [30] (Fig. 2A). For benchmarking, the spectroscopic in vitro properties of CaNeon and mCyRFP1-CaNeon were compared to the established Ca^2+^ sensor GCaMP6f [44]. The extinction coefficient, quantum yield and Ca^2+^ sensitivity of both indicators (Supplementary Fig. 1D) were similar to that of GCaMP6f but with better linearity (Hill coefficient 1.5–1.6 instead of 2.3).

Taking into account our previous experience in mice [10], when preparing miR9-regulated lentiviral vectors encoding mCyRFP1-CaNeon we switched to a stronger promotor (CMV instead of PGK; Fig. 2C vs. Fig. 1B). From such a vector, the sensor expressed well both in Human Embryonic Kidney (HEK) 293 cells in culture (Supplementary Fig. 1E–H) and human cortical microglia in organotypic slices (Fig. 2D–F). For initial testing of the new construct, HEK 293 cells were stimulated by a bath application of caffeine (Supplementary Fig. 1F–H), an agonist of ryanodine receptors releasing Ca^2+^ from the intracellular Ca^2+^ stores [45], and microglia – by a local pressure application (250 ms, 12 psi) of the purinergic receptor agonist ATP (Fig. 2D), resulting in both cases in large Ca^2+^ transients.

The specificity of the miR9-regulated microglial labeling was assessed in fixed organotypic slices, labeled with antibodies against a microglia/macrophage-specific marker Ionized Ca^2+^-binding adaptor molecule 1 (Iba-1) and mCherry, because mCherry has 63% homology with mCyRFP1 and 33% homology with CaNeon, thus enabling good visualization of mCyRFP1-CaNeon (Fig. 2E). In fixed tissue, the native fluorescence of CaNeon was too low to be visible in the green channel. Our transfection protocol (1:1000 virus dilution in hCSF) enabled the expression of mCyRFP1-CaNeon in 52.38 ± 0.9% (mean ± SEM) of Iba-1-positive cells (316 cells/23 fields of view (FOVs)), while 91.67 ± 32.29% (254 cells/41 FOVs) of mCyRFP1-CaNeon-positive cells were also Iba-1-positive (Fig. 2F). In contrast to our previous experiments in mice [10], in the human brain we did identify another weakly-labeled Iba-1-negative population of spindle-shaped cells, often associated with blood vessels (presumed pericytes, arrows in Fig. 2E and Supplementary Fig. 3). The latter, however, were dim and therefore hardly visible in situ and were morphologically too different to be mistaken for microglia. These cells were excluded from further analyses.

Taken together, the novel technique described above enables real-time morphological and functional analyses of human microglia, including longitudinal analyses of process/cell body motility (Supplementary Movie 1) as well as Ca^2+^ imaging.

### Morphotype-specific properties of microglial Ca^2+^ signals

In contrast to the mouse cerebral cortex, in which under homeostatic conditions more than 90% of microglia have ramified morphology, in the human postmortem cortex microglial cells appear much more heterogeneous, with only half of the population showing ramified morphology and another half being composed of hypertrophic (also called reactive; 32%) and ameboid (18%) cells [21]. Consistently, out of 81 cells analyzed in this study, 46.91% were ramified, 30.86% hypertrophic and 22.22% ameboid (Fig. 3A). All 3 microglial morphotypes were also present in freshly resected human cortical tissue (Supplementary Fig. 4). Although at the first glance the density of microglia, the median distance to the nearest neighbor, the mean cell diameter and the soma shape in freshly resected and organotypically cultured slices looked similar, a detailed examination revealed significant differences between the two groups for all but last parameters (Supplementary Fig. 4). Besides, the parameters related to cell diameter (the cell surface area and cell volume) also differed between the groups (Mann–Whitney Test, P < 10^–4^ for both comparisons). The median distance to the nearest neighbor was 30.5 for acute and 45.2 for cultured slices, with the latter being much closer to the in vivo mouse data (~ 50 µm, [46]). This is surprising, as the density of microglia was reported to be higher in the mouse compared to the human cortex [24].

Different morphotypes differed in terms of their Ca^2+^ signaling. Like their mouse counterparts in vivo [7, 8], human microglia in organotypic slices showed ongoing Ca^2+^ signaling (Fig. 3A). While the fractions of active cells (showing at least one Ca^2+^ transient at any subcellular location during the 15-min-long recording period) were approximately similar among ramified and hypertrophic microglia (Fig. 3B), the patterns of Ca^2+^ transients sampled over the entire cell looked different, with an increase in the amplitude and/or frequency of these transients from ramified to ameboid microglia and a significant reduction in the fraction of active cells for ameboid microglia (Fig. 3A-B; P = 3*10^–4^, Chi-squared test). Moreover, the median basal ratios (proportional to the basal [Ca^2+^]_i_) of active cells increased gradually from ramified through hypertrophic to ameboid cells, with the difference between the ramified and ameboid cells reaching the level of statistical significance (Fig. 3C; P = 1.2*10^–2^, Kruskal–Wallis test). In 3 hypertrophic and 1 ameboid cell, ongoing Ca^2+^ transients showed oscillatory behavior (e.g., ameboid Cell 1 in Fig. 3A) with a median frequency of 12.33*10^–3^ s^−1^. In 4 recorded cells the microglial processes formed structures, reminiscent of phagocytic cups, with cups often showing compartmentalized Ca^2+^ transients (Supplementary Movie 2). Although we did not observe any somatic movements over a 15-min-long recording period, many ramified and hypertrophic cells showed vivid process motility, also often associated with compartmentalized Ca^2+^ transients (Supplementary Movie 1).

Therefore, the subsequent analyses of Ca^2+^ signaling were conducted based on active subcellular compartments (regions of activity, ROA) using the Begonia ROA detection algorithm [39]; see Materials and methods for details). On average, individual microglial cells contained 2.53 ± 0.35 (mean ± SEM; n = 56 cells) active subcellular compartments, some of which are exemplified in Fig. 4A. Out of 78 ROA detected in ramified cells, 75.6% were localized to the processes, 11.5% covered soma and processes and 12.8% covered cell somata only (Fig. 4B). Surprisingly, we have rarely observed global Ca^2+^ signals invading the entire cell. The spatial distribution of Ca^2+^ signals in hypertrophic cells was similar to that in ramified microglia (P = 0.31, Chi-squared test). In ameboid cells, however, the spatial pattern of Ca^2+^ signals was entirely different, with 66.7% of ROA localized to cell somata (P < 10^–3^, Chi-squared test). Consistently, the areas of individual ROAs were small in ramified, somewhat larger and heterogeneous in hypertrophic and significantly larger in ameboid cells (P = 2.3*10^–3^, Kruskal–Wallis test; Fig. 4C), whereas the morphotype-specific frequencies of ongoing Ca^2+^ signals were similar (Fig. 4D). Next, we characterized the properties of Ca^2+^ transients derived from different ROA in the 3 morphotypes (Fig. 4E–H). The median amplitudes of Ca^2+^ transients were significantly smaller in hypertrophic, compared to ramified and ameboid, microglia (Fig. 4F). The durations (full width at half maximum, FWHM) were larger and the total Ca^2+^ load (area under the curve, AUC) was smaller in hypertrophic compared to ramified cells (Fig. 4G, H).

Detailed analyses of individual Ca^2+^ transients revealed that although the majority of ROAs in ameboid cells were located in cell somata (Fig. 4B), the soma and process ROAs showed many more Ca^2+^ transients (Fig. 5A). Moreover, for ramified and hypertrophic morphotypes process Ca^2+^ transients had the highest amplitudes and AUCs, whereas their durations varied between the morphotypes (Supplementary Fig. 5). In ramified microglia, the durations of process Ca^2+^ transients were the shortest (Supplementary Fig. 5B), whereas in hypertrophic and ameboid microglia the durations of Ca^2+^ transients localized to soma and processes became gradually longer than that of somatic Ca^2+^ transients, reaching the level of significance in ameboid microglia (Supplementary Fig. 5E and H). The data also revealed the compartment-specific heterogeneity of Ca^2+^ transients in ramified, hypertrophic and ameboid microglia and prompted the compartment-specific comparison across morphotypes (Fig. 5B–J). In general, somatic Ca^2+^ transients of ameboid cells had the largest amplitude and the shortest duration (Fig. 5B, C), whereas ramified cells had the largest AUCs (Fig. 5D). For ROA located in soma and processes, the amplitudes and AUCs increased gradually from ramified through hypertrophic to ameboid cells (Fig. 5E and G). The process Ca^2+^ transients had higher amplitudes and shorter durations in ramified microglia (Fig. 5H, I).

Thus, our data revealed the marked compartmentalization of Ca^2+^ transients in human microglia. Interestingly, the basic properties of these Ca^2+^ transients differed dramatically across the compartments as well as morphotypes.

## Discussion

**Fig. 6:**
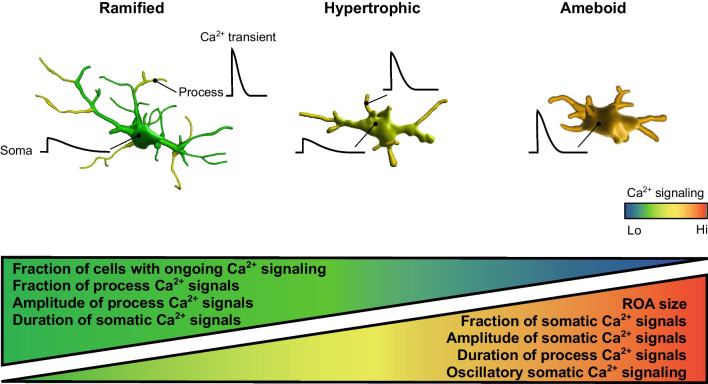
Ongoing Ca^2+^ signals in human microglia are subcompartment- and morphotype-specific. Graphical summary of main findings. See discussion for details

In this study, we introduced a new approach for functional analyses of in situ human microglia cultured in hCSF and applied it to characterize the ongoing Ca^2+^ signaling in microglial morphotypes (i.e., ramified, hypertrophic and ameboid microglia), present in the human brain. Our data revealed distinct functional properties of each morphotype. Whereas in ramified microglia basal [Ca^2+^]_i_ was low and ongoing Ca^2+^ transients were mostly compartmentalized in microglial processes, having large amplitudes and short durations, the ameboid microglia had significantly higher basal [Ca^2+^]_i_ and the vast majority of large amplitude Ca^2+^ signals invaded cell somata. Moreover, the fraction of cells with ongoing Ca^2+^ signaling, the fraction and the amplitude of process Ca^2+^ signals and the duration of somatic Ca^2+^ signals decreased when moving along the microglia activation pathway, i.e. from ramified via hypertrophic to ameboid microglia (Fig. 6). In contrast, the size of active compartments, the fraction and the amplitude of somatic Ca^2+^ signals and the duration of process Ca^2+^ signals increased with microglial activation (Fig. 6). While imaged microglial cells often showed overt process movement associated with localized Ca^2+^ signals, within the framework of the present study (15-min-long recording period) we have not observed any translocations of cell somata.

To enable Ca^2+^ imaging of human microglia, we combined several cutting-edge techniques, recently developed by our laboratories. Thus, organotypic brain slices derived from fresh human biopsies were cultured in human CSF. This approach preserves well not only neurons [25, 26] but also different morphotypes of human microglia (Fig. 1). Contrary to in vivo mouse cortex with or without transplanted human cortical organoids, which contains ramified human or mouse microglia [46–48], and cultured human iPSC-derived microglial cells, which are largely ameboid [16–20], our model harbors all morphotypes, thus allowing their comparative study. Moreover, throughout the experiment, the microglia dwell in their native microenvironment. This is important because of the well-known environment-induced cell-non-autonomous shifts in microglial phenotype [13, 48]. The distribution of ramified, hypertrophic and ameboid microglia in our preparation was close to that found in fresh-frozen postmortem cortical samples [21, 22]. Accordingly, we have also found all 3 morphotypes in acutely resected human cortical tissue.

The use of RGB labeling provides each microglial cell with a unique color tag and enables the longitudinal monitoring of its behavior, including cell death or division, phagocytosis, somatic dislocation or process movement under control conditions and, in the future, also in human disease models [49]. Finally, the development of a novel genetically encoded Ca^2+^ indicator mCyRFP1-CaNeon, well-suited for single- as well as two-photon-based ratiometric Ca^2+^ imaging, adds to the versatile microglia analyses toolbox and has enabled us to measure the morphotype-specific basal Ca^2+^ levels. Compared to popular GCaMPs, CaNeon employs a brighter fluorophore (mNeonGreen versus GFP) and has only 2 (vs. 4) Ca^2+^ binding sites, thus providing better linearity of the sensor and reducing the overall burden of Ca^2+^ buffering. Furthermore, it enables ratiometric Ca^2+^ imaging in conditions of one-photon excitation (e.g. a ratiometric miniscope-based (deep-brain) Ca^2+^ imaging in freely moving animals [50, 51]).

Careful identification of all active pixels within the cell (Fig. 4), allowed us to analyze the spatial dimension of microglial Ca^2+^ signaling. The data revealed that microglial Ca^2+^ signals are highly compartmentalized and rarely spread over the entire cell. Moreover, in ramified microglia, the amplitudes and time courses of Ca^2+^ transients differed significantly between different subcompartments (Fig. 6), with large and short process Ca^2+^ transients likely targeting different signaling pathways compared to small but prolonged somatic Ca^2+^ signals [52, 53].

This study uniquely analyzed Ca^2+^ signaling in microglia stemming from decades-old brains, in sharp contrast to recent analyses of iPSC-derived microglia, generated, on average, 30–50 days before the experiment [17, 19, 20]. Consistently, the properties of the two cell populations differed substantially both in morphology (largely ameboid iPSC-derived microglia vs. 3 different morphotypes analyzed in our study) and function. While iPSC-derived microglia moved vividly around the dish with the mean speed of 5 µm/min [17, 19], we did not observe any translocation of microglial somata over the 15-min-long recording period, reminiscent to in vivo data from adult mice [34, 54, 55]. Consistent with their morphology and similar to our ameboid cells, iPSC-derived microglia showed global Ca^2+^ signals invading both somata and processes [20], quite in contrast to much more compartmentalized Ca^2+^ signaling patterns of ramified and hypertrophic microglia studied here. It also has to be kept in mind that in contrast to iPSCs, microglia stem from the extra-embryonic yolk sack macrophages [56].

So far, Ca^2+^ signaling of ramified microglia was exclusively studied in mice under in situ [57, 58] or in vivo [6–10, 59] conditions. However, recent comparative mouse-human studies revealed several functional differences between the human and mouse microglia. First, human microglia renew at a median rate of 28% per year [23], which means that some microglia dwell in the human brain for more than two decades, whereas mice live ~ 2 years and the oldest mouse microglia in which Ca^2+^ signaling was studied were 18–21-month-old [60]. Secondly, despite the overall similarity between the genes expressed by human and mouse microglia, several immune genes do not have a clear 1:1 mouse ortholog (e.g., complement receptor type 1, HLA-DRB1, HLA-DRB5, TLR, Fcγ and SIGLEC receptors) or display < 60% identity between human and mouse at the primary amino acid sequence (e.g. TREM2), and several rodent gene modules, including complement, phagocytic and neurodegeneration-susceptibility genes, differed substantially from primate microglia [14, 47, 61]. Moreover, whereas in most species microglia showed a single dominant transcriptional state, human microglia displayed substantial heterogeneity [61] and, for example, responded to neurodegeneration and Alzheimer’s disease pathology by upregulation of TMEM119, P2RY12 and CX3CR1 genes, contrary to their downregulation in mouse models of the disease [62, 63].

Against this backdrop, we have compared the ongoing Ca^2+^ signaling in human and mouse microglia and have spotted several major similarities. First, the studied human cells seemed to belong to a continuum of microglial functional states with ramified and ameboid microglia building the two extremes and hypertrophic microglia spreading among multiple intermediate states [64]. Moreover, (i) the basal [Ca^2+^]_i_ increased with the degree of activation both in human (Fig. 3C) and mouse [10] microglia; (ii) both species showed spontaneous ongoing Ca^2+^ signaling; (iii) microglial Ca^2+^ transients in both species were highly compartmentalized (Fig. 4; [5, 6]) and (iv) the spatial distribution of ongoing Ca^2+^ signals switched from process- to soma-dominated patterns during microglial activation (Figs. 3, 4, 5; [6, 7, 9]).

### Limitations of the study

Analyses of the resected human tissue provide a unique opportunity to study decades-old human microglia in their native microenvironment. However, because this tissue stems from epilepsy or tumor surgeries it cannot a priori be considered normal. In the current study, the postoperative histologic examination of the resected tissue was only done for the epilepsy patient (Table 1, patient 5), showing no evidence of focal cortical dysplasia or other pathology. In the case of the intraventricular glioneuronal tumor (Table 1, patient 6), the resected and the tumor tissues were far (~ 2 cm) apart, suggesting that the resected access tissue was healthy. In all other cases resected and tumor tissues were located closer. Of note, electrophysiological and plasticity properties of temporal cortex pyramidal cells from healthy rats and patient epilepsy/tumor resection tissues were shown to be similar [65]. Recently, the same was shown to be true for basic electrophysiological properties of healthy mouse microglia and human microglia stemming from epilepsy resection tissue [66]. We cannot exclude, however, that other properties of analyzed microglia might have been modified by the preexisting brain pathology.

Furthermore, expression of the genetically encoded Ca^2+^ indicator in microglia requires organotypic slice culturing, which might also change their functional properties. This drawback our approach shares with all other available techniques analyzing Ca^2+^ signaling in human microglia [15–20]. Organotypic slice culturing in hCSF was established several years ago, and our previous data revealed striking similarities between the properties of pyramidal cells and interneurons recorded in acute or organotypically cultured (for 7–14 days) slices [25, 26]. Both cell types showed stable action potential (AP) firing frequencies, the AP half widths, resting membrane potentials, sag potential amplitudes, input resistances as well as cell-type specific firing properties (e.g. spike frequency adaptation in pyramidal cells). Moreover, reconstructions of 24 pyramidal cells cultured for 0–14 days revealed a surprising stability of their morphological features, including dendritic spines, pointing to the absence of overt inflammation. Finally, the high-density microelectrode array recordings showed that hypersynchronous or epileptiform-like activity is not typical for this kind of preparation [28, 67]. Concerning microglia, a comparison of freshly resected and hCSF-cultured tissue (Supplementary Fig. 4) revealed that although the cell density, median distance to the nearest neighbors, mean microglial cell diameter, volume, surface area, and soma shape (sphericity) were within the similar range, a detailed examination revealed significant differences between the cells of the two groups for all but last parameters. Although the compared tissue stemmed from different donors, the results were consistent within the groups suggesting that the observed difference is due to slice culturing rather than inter-individual or surgery-related variability. Thus, although organotypic slice culturing in hCSF prevents the development of overt inflammation and preserves the in vivo-like distribution of microglial morphotypes, some differences in functional properties between in situ and in vivo microglia can't be excluded.

## Conclusions

Our data establish biopsy-derived organotypic human brain slices, cultured in human CSF, as a valid in situ model for analyses of human microglia thus increasing the versatility of the available toolbox. This model provides a unique possibility to study adult, decades-old microglia in their natural microenvironment and reveals that many functional properties of these cells, including vivid process motility and compartmentalized ongoing Ca^2+^ signaling, the pattern of which is morphotype-specific, are reminiscent of that seen in vivo in rodent microglia. The latter cells, however, are ramified under homeostatic conditions, while much longer-lived human microglia express a continuum of different morphotypes.

### Supplementary Information

Supplementary Material 1.

Supplementary Material 2.

Supplementary Material 3.

## Data Availability

All relevant data are included in the manuscript and supplementary files. Any further inquiries can be directed to the corresponding author.
