# Supplementary material for: Morphotype-specific calcium signaling in human microglia
Source: J Neuroinflammation. 2024 Jul 17;21:175. doi: 10.1186/s12974-024-03169-6 (PMC11256502; doi:10.1186/s12974-024-03169-6)
Supplement: Supplementary file 1 — Supplementary Material 1. [file 12974_2024_3169_MOESM1_ESM.pdf]

Supplementary materials for

Morphotype-specific calcium signaling in human microglia

Sofia Nevelchuk[1], Bianca Brawek[1], Niklas Schwarz[2], Ariel Valiente-Gabioud[3], Thomas V. Wuttke[2,4], Yury Kovalchuk[1], Henner Koch[5], Anke Höllig[6], Frederik Steiner[1], Katherine Figarella[1#], Oliver Griesbeck[3], Olga Garaschuk[1*]

[1]Institute of Physiology, Department of Neurophysiology, Eberhard Karls University of Tübingen, Tübingen, Germany
[2]Department of Neurology and Epileptology, Hertie Institute for Clinical Brain Research, University of Tübingen, Tübingen, Germany
[3]Tools for Bio-Imaging, Max-Planck-Institute for Biological Intelligence, Martinsried, Germany
[4]Department of Neurosurgery, University of Tübingen, Tübingen, Germany
[5] Department of Epileptology, Neurology, RWTH Aachen University Hospital, Aachen, Germany
[6] Department of Neurosurgery, RWTH Aachen University, Aachen, Germany

[#]present address: Department of Anesthesiology, Critical Care and Pain Medicine. University of Texas Health Science Center at Houston, Houston, TX, USA

*Corresponding author
Correspondence should be addressed to:
Prof. Dr. Olga Garaschuk
Institute of Physiology, Department of Neurophysiology,
University of Tübingen
Keplerstr. 15, 72074 Tübingen, Germany
Tel: +49-07071 29 73640
Fax: +49-07071 29 5395
E-mail: olga.garaschuk@uni-tuebingen.de

Supplementary figures

Supplementary Figure 1. **Spectroscopic *in vitro* properties of CaNeon and mCyRFP1-CaNeon.** (**A**) The amino acid sequence of CaNeon: mNeonGreen (green), TnC_{min} (orange), linkers (black). The amino acid changes introduced during sensor optimization and thus differing from parental NRS1.2 are underlined. (**B-C**) Emission spectra of CaNeon (**B**) and mCyRFP1-CaNeon (**C**) at increasing concentrations of Ca^{2+} (from light to dark green: 0, 0.027, 0.065, 0.1, 0.225, 0.361, 0.602, 0.853, 1.73, 2.85, 7.37 and 14.9 mM). (**D**) Summary of the main spectroscopic parameters of both new Ca^{2+} indicators (see Materials and methods for measurement details) in comparison to those of the commonly used Ca^{2+} indicator GCaMP6f [1]. (**E-H**) Ca^{2+} responsiveness of mCyRFP1-CaNeon in HEK 293 cells, cultured using the standard protocol [2] and transduced with miR9-regulated lentiviral vectors. Average intensity images of a sample FOV recorded in green (**E**, left) and red (**E**, right) channels, and their overlays (**F**), taken at different time points (t1 and t2, see also (**G-H**)) during the measurement, illustrate the baseline level of CaNeon and mCyRFP1 fluorescence (**E** and **F**, left) and fluorescence changes (**F**, right) caused by a 1-min-long bath application of 20 mM caffeine, dissolved in the Ringer's solution. The ΔF/F and ΔR/R signals recorded in regions of interest delineated in (**F**, left) are shown in (**G**) and (**H**), respectively.

Supplementary Figure 2. **ROA detection and analysis pipeline.** (**A**) Schematic representation of the active voxel detection algorithm in the MATLAB Begonia framework. Right panel: schematic overlay of the binary array of active voxels per each frame of the cell recording. (**B**) Summed (over time) image of all active pixels in this recording. (**C**) Averaged intensity projection of the recorded cell with the Otsu threshold

applied to segment the soma (in red) from the background. (**D**) An overlay of non-overlapping ROAs identified as described in Materials and methods (cyan, green and pink colors) and the somatic area (red) defined based on the Otsu threshold.

Supplementary Figure 3. miR-9 regulated lentiviral vectors weakly label a non-microglial population of human cells. (**A**) MIP (4-16 μm depth) image of a fixed human organotypic brain slice, labeled with antibodies against a microglia/macrophage marker Iba-1 (magenta) and mCyRFP1-CaNeon (green). (**B**) MIP (13-21 μm depth) image of a fixed cortical slice of a mouse mutant expressing DsRed under the NG2 promotor, thus labeling NG2-positive oligodendrocyte precursor cells and pericytes [3]. The tissue was labeled with antibodies against RFP (green, recognizing DsRed) and NG2 cell marker PDGFRα (magenta). Note the similarity in morphology between the weakly-labeled non-microglial human cells (**A**, arrows) and mouse pericytes (**B**, arrows).

Supplementary Figure 4. Morphology of microglia from freshly resected human tissue. (**A**) Overview MIP images (5-15, 4-11, 17-26 μm depth, respectively) of the freshly resected human cortical tissue, labeled with antibodies against Iba-1. Yellow-reddish structures likely represent the autofluorescent wear-and-tear pigment lipofuscin, known to have broad excitation and emission spectra [4]. Arrow and arrowhead exemplify cells, which we have scored as hypertrophic and ameboid throughout the experiments. (**B**) Sample zoomed-in MIP images of individual ramified, hypertrophic and ameboid microglia (1-13, 31-37, 34-39 μm depth, respectively). (**C-F**) Box plots, illustrating the distributions of cell density (**C**, 61 and 56 3D stacks, respectively), median distance to the nearest neighbor (**D**, 56 and 55 3D stacks,

respectively) as well as mean cell diameter (**E**) and sphericity (**F**) of microglial somata from freshly resected (n=175 cells) and cultured (n=55 cells) human tissue. Mann-Whitney Test, $P<10^{-4}$ (**C-E**) and P=0.52 (**F**).

Supplementary Figure 5. Characteristics of morphotype-specific microglial Ca^{2+} transients in different subcellular compartments. (**A-C**) Box plots showing the amplitude (**A**; $P=6.5*10^{-10}$, $4.8*10^{-14}$ and $5.2*10^{-3}$ for comparison of processes to soma and processes, processes to soma and soma and processes to soma, respectively, in ramified microglia (here and below Kruskal-Wallis test followed by Holm-Bonferroni post hoc test for multiple comparisons)), FWHM (**B**; $P=4*10^{-3}$ for comparison of microglial processes to soma), and AUC (**C**; $P=8.7*10^{-9}$, $7.4*10^{-10}$ and $3.4*10^{-4}$ for comparison of processes to soma and processes, processes to soma and soma and processes to soma, respectively, in ramified microglia) of Ca^{2+} transients recorded in different subcellular compartments of ramified microglia. (**D-F**) Box plots showing the amplitude (**D**; $P=2.4*10^{-8}$, $9.2*10^{-6}$ and 0.05 for comparison of processes to soma and processes, processes to soma and soma and processes to soma, respectively), FWHM (**E**), and AUC (**F**; $P=1.4*10^{-9}$, $2.4*10^{-6}$ and 0.02 for comparison of processes to soma and processes, processes to soma and soma and processes to soma, respectively) of Ca^{2+} transients recorded in different subcellular compartments of hypertrophic microglia. (**G-I**) Box plots showing the amplitude (**G**; $P=2.8*10^{-3}$), FWHM (**H**; $P=1.4*10^{-3}$), and AUC (**I**; $P=1.1*10^{-4}$) of Ca^{2+} transients recorded in soma and processes as well as soma of amoeboid microglia.

Supplementary Movie 1. Process motility of human microglia. An overlay of CaNeon (green) and mCyRFP1 (red) channels, showing a microglial cell, vividly

moving its processes. Each channel is an average intensity projection of 6 images (10-16 µm, step 1 µm). Note several increases in green fluorescence (Ca^{2+} transients) accompanying process movement. Scale bar: 10 µm. Frame rate: 4.57 s, the movie plays 100 times faster.

Supplementary Movie 2. Localized Ca^{2+} signaling in phagocytic cups. An overlay of CaNeon (green) and mCyRFP1 (red) channels, showing a microglial cell with several phagocytic cups. Each channel is an average intensity projection of 9 images (16-24 µm, step 1 µm). Note several increases in green fluorescence (Ca^{2+} transients) localized in the cup vicinity (arrowheads) and one cup, which is pulled towards the parent process (arrow). Scale bar: 10 µm. Frame rate: 4.94 s, the movie plays 100 times faster.

References

[1] T.W. Chen, T.J. Wardill, Y. Sun, S.R. Pulver, S.L. Renninger, A. Baohan, E.R. Schreiter, R.A. Kerr, M.B. Orger, V. Jayaraman, L.L. Looger, K. Svoboda, D.S. Kim. Ultrasensitive fluorescent proteins for imaging neuronal activity. *Nature* 2013,499(7458):295-300.

[2] R. Loew, Y. Meyer, K. Kuehlcke, L. Gama-Norton, D. Wirth, H. Hauser, S. Stein, M. Grez, S. Thornhill, A. Thrasher, C. Baum, A. Schambach. A new PG13-based packaging cell line for stable production of clinical-grade self-inactivating gamma-retroviral vectors using targeted integration. *Gene Ther* 2010,17(2): 272-280.

[3] X.Q. Zhu, D.E. Bergles, A. Nishiyama. NG2 cells generate both oligodendrocytes and gray matter astrocytes. *Development* 2008,135(1):145-157.

[4] G. Eichhoff, M.A. Busche, O. Garaschuk. In vivo calcium imaging of the aging and diseased brain. *Eur J Nucl Med Mol Imaging* 2008, 35:S99-106.

A

10 20 30 40 50 60
MVSKGEEDNMGSLPATHELHIFGSINGVDFDMVGQGSGNPNDGYEELNLKSTKGDLQFSP
70 80 90 100 110 120
WILVPHIGYGFHQYLPYPDGMSPFQAAMVDGSGYQVHRTMQFEDGASLTVNYRYTYEGSH
130 140 150 160 170 180
IKGEAQVKGTGFPADGPVMTNSLTAADLGWDSEEELSEYFRIFDFDGNGFIDREEFGDII
190 200 210 220 230 240
RLTGEQLTDEDVDEIFGGSDTDKNGRIDFDEFLKMVENVQLTDNNRSKKTYPNDKTIIST
250 260 270 280 290 300
YKWSYTTDNGKRYRCTARTTYTFAKPMAANYLKNQPMYVFRKTELKHSKTELNFKEWQKAFTD

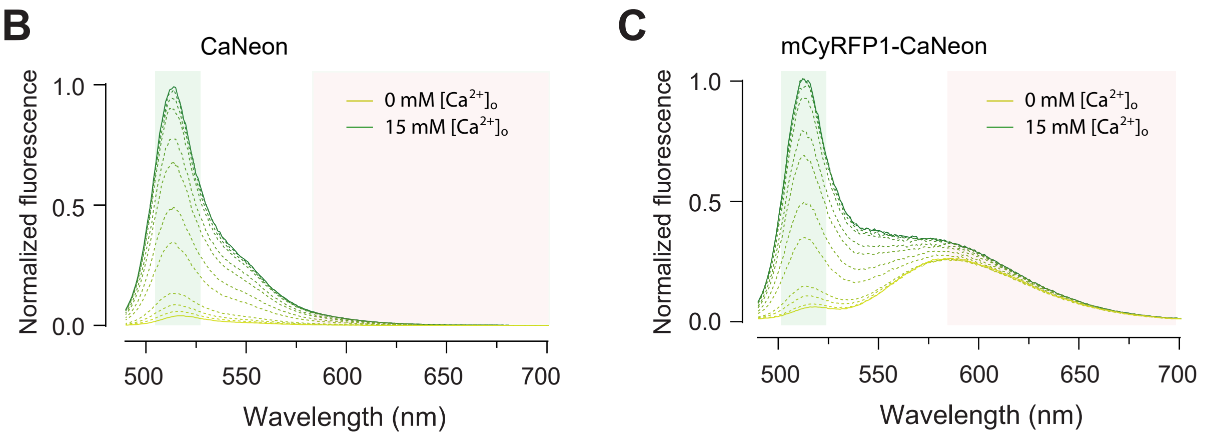

D

	$\Delta F/F_0$	$\Delta R/R_0$	EC ($mM^{-1}cm^{-1}$)	QY	Brightness	K^*_{obs} (s^{-1})	Kd (nM)	Hill coeff.	K_{off} (s^{-1})	pKa
CaNeon	30		74	0.6	46	4.9	385	1.5	1.7	6.5
mCyRFP1-CaNeon		16	70	0.6	43	4.2	401	1.6	1.7	6.6
GCaMP6f	52		62	0.6	37	n.d.	375	2.3	3.9	8.8

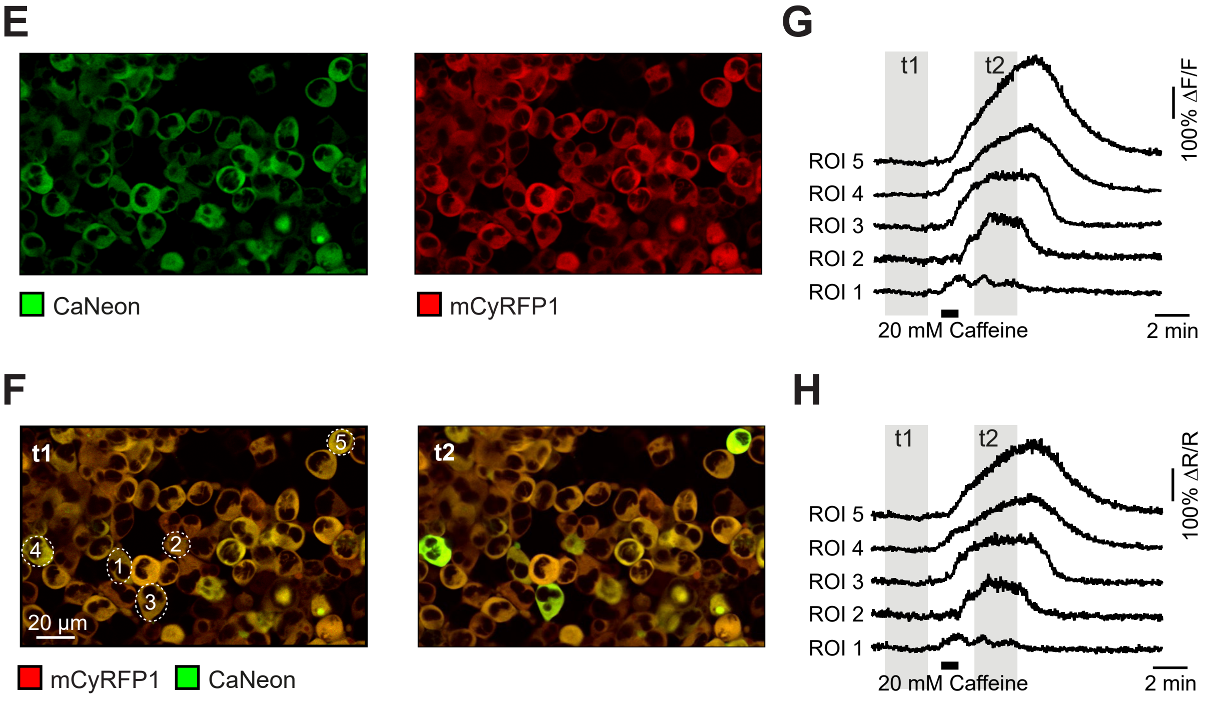

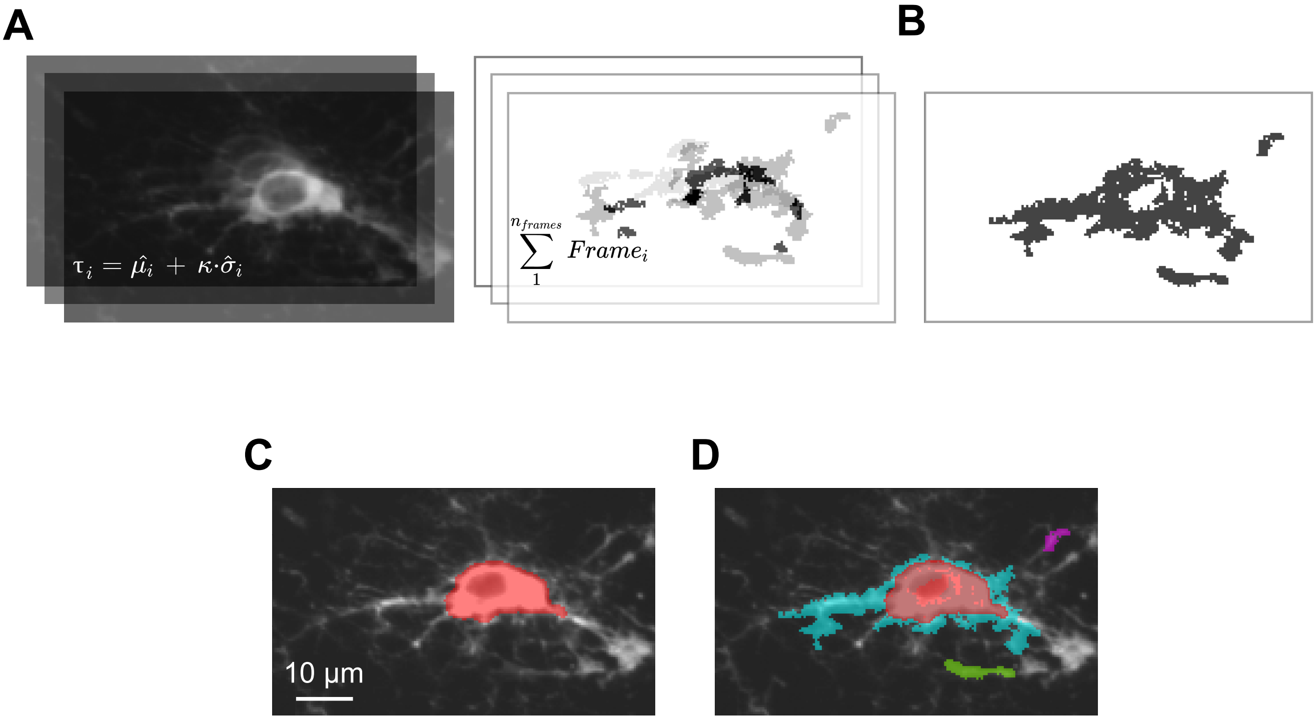
A
B
C
D
$\tau_i = \hat{\mu}_i + \kappa \cdot \hat{\sigma}_i$
$\sum_{1}^{n_{frames}} Frame_i$
10 μm

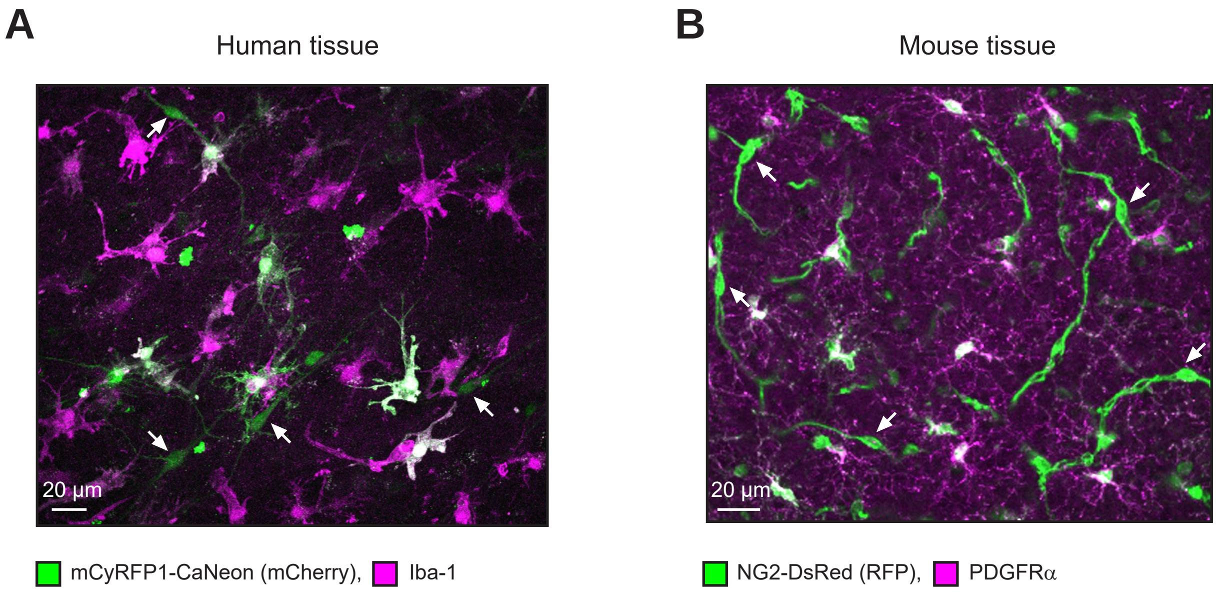

A
Human tissue
20 µm
mCyRFP1-CaNeon (mCherry),
Iba-1
B
Mouse tissue
20 µm
NG2-DsRed (RFP),
PDGFRα

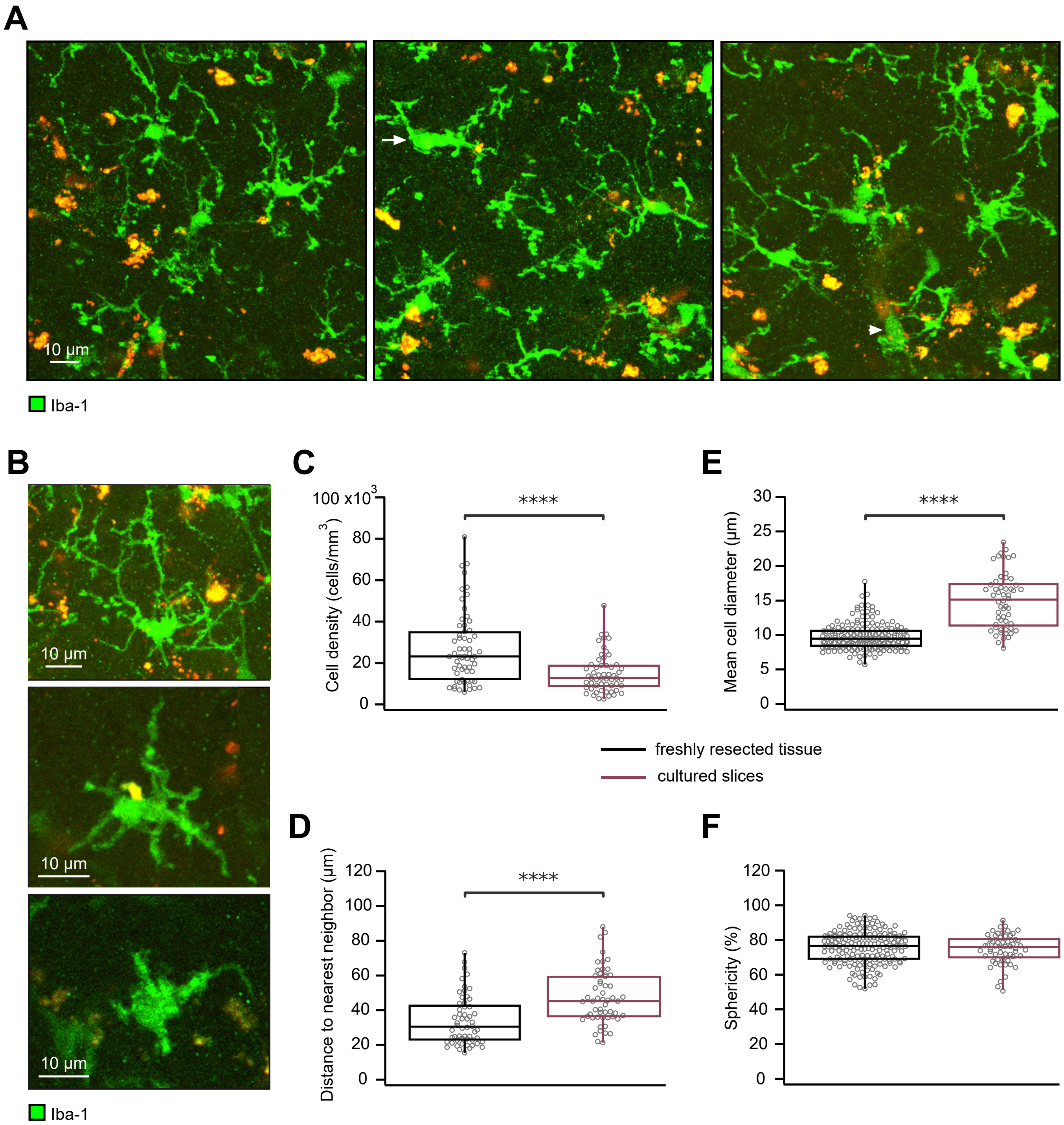
A
10 µm
Iba-1
B
10 µm
10 µm
10 µm
Iba-1
C
100 x10^3
80
60
40
20
0
Cell density (cells/mm^3)
****
E
30
25
20
15
10
5
0
Mean cell diameter (µm)
****
freshly resected tissue
cultured slices
D
120
100
80
60
40
20
0
Distance to nearest neighbor (µm)
****
F
120
100
80
60
40
20
0
Sphericity (%)

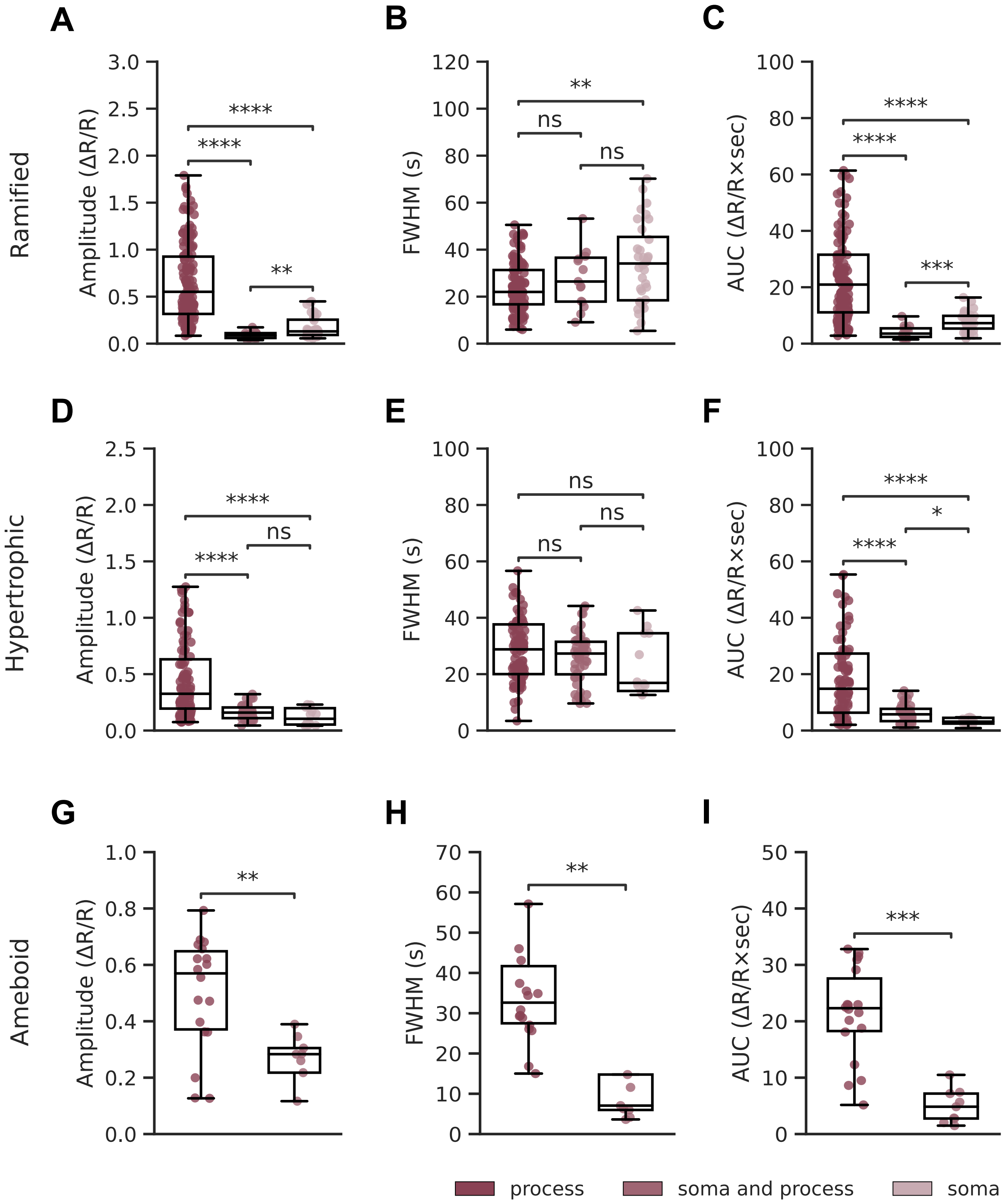
A
B
C
Ramified
Amplitude (ΔR/R)
FWHM (s)
AUC (ΔR/R×sec)
****
**
***
ns
D
E
F
Hypertrophic
*
G
H
I
Ameboid
process
soma and process
soma
